# Intraductal magnetic resonance imaging of cholangiocarcinoma - a practical possibility

**DOI:** 10.3389/fonc.2024.1306242

**Published:** 2024-04-08

**Authors:** Richard R. A. Syms, Christopher A. Wadsworth, Evdokia Kardoulaki, Attapol Titapun, Wuttisak Boonphongsathien, Prakasit Sa-Ngiamwibool, Shuo Zhang, Simon D. Taylor-Robinson, Nittaya Chamadol, Watcharin Loilome

**Affiliations:** ^1^ Department of Electrical and Electronic Engineering, Imperial College London, London, United Kingdom; ^2^ Department of Surgery and Cancer at St Mary’s Hospital, Imperial College London, London, United Kingdom; ^3^ Department of Surgery, Faculty of Medicine, Khon Kaen University, Khon Kaen, Thailand; ^4^ Department of Radiology, Faculty of Medicine, Khon Kaen University, Khon Kaen, Thailand; ^5^ Department of Pathology, Faculty of Medicine, Khon Kaen University, Khon Kaen, Thailand; ^6^ Health Systems, Clinical Science, Philips Healthcare Germany, Hamburg, Germany; ^7^ Cholangiocarcinoma Research Institute, Faculty of Medicine, Khon Kaen University, Khon Kaen, Thailand

**Keywords:** cholangiocarcinoma, intraductal MRI, liver fluke, T2 mapping, tumor early detection

## Abstract

Intraductal T2 mapping based on a catheter receiver is proposed as a method of visualizing the extent of intraductal and periductal cholangiocarcinoma (CCA). Compared to external receivers, internal receivers provide locally enhanced signal-to-noise ratios by virtue of their lower field-of-view for body noise, allowing smaller voxels and higher resolution. However, inherent radial sensitivity variation and segmentation for patient safety both distort image brightness. We discuss simulated T2 weighted images and T2 maps, and *in vitro* images obtained using a thin film catheter receiver of a freshly resected liver specimen containing a polypoid intraductal tumor from a patient with CCA. T2 mapping provides a simple method of compensating non-uniform signal reception patterns of catheter receivers, allowing the visualization of tumor extent without contrast enhancement and potentially quantitative tissue characterization. Potential advantages and disadvantages of *in vivo* intraductal imaging are considered.

## 1 Introduction

Cholangiocarcinoma (CCA) is rare in Europe and North America, where known risk factors include primary sclerosing cholangitis (PSC), viral hepatitis, and occupational exposure to industrial chemicals (1). Presentation is typically late, when disease is advanced. The 5-year survival is consequently low, and surgical resection is the only real curative possibility, although recently approved targeted chemotherapy may offer some hope in a minority of patients (1, 2). The incidence of CCA in South-East Asia is around 100 times higher than Western Europe, due to consumption of raw and partly cooked fish, contaminated with the parasitic liver flukes, *Opisthorchis viverrini* or *Clonorchis sinensis* (1). Around 60 million people in the region are at risk of infection, with CCA a possible consequence for 1-2% of fluke-infected individuals (3). Flukes can be eradicated using anthelmintic drugs, but re-infestation is common and food safety education provides only a partial solution (3).

Risk groups are screened in Thailand, using stool analyses and urinary dipsticks to detect fluke infestation and ultrasound to locate periductal fibrosis and mass-forming tumors (4). Similar programs are now being developed in Lao PDR. Magnetic resonance imaging (MRI) and computed tomography (CT) are used for confirmatory diagnosis. CCA is heterogeneous, and can present an extrahepatic and intrahepatic tumor, with mass-forming, periductal infiltrating and intraductal sub-types (5). MRI can delineate obstructed bile ducts owing to the long T2 time constant of bile, and contrast-enhancement can highlight mass-forming tumors due to their enhanced micro-vascularity (6). However, the small duct wall changes in early-stage disease are hard to detect (7), so precise staging and surgical planning are difficult.

Here, we make the case for intraductal MR imaging with T2 mapping to improve tumor visualization for effective patient management. These strategies are not only applicable in Thailand where CCA is a common problem, but potentially to all healthcare systems where a hepatobiliary service is offered.

## 2 The clinical case for improved imaging

Except in South-East Asia, CCA remains a rare cancer, but its incidence has been rising for the past 30 years, with significant geographical variations (8). North-East Thailand has by far the highest age-standardized incidence rate (ASIR) of 85/100000 (8). By comparison, the United States has an ASIR of 1.6/100000 and the United Kingdom 2.2/100000 (8), which are more in keeping with the world average.

The early detection of CCA remains difficult (9). Guidelines recommend standard contrast-enhanced CT or MRI for the diagnosis and staging of CCA. Both can identify mass-forming tumors, whereas the periductal infiltrating form is difficult to detect. When complicating biliary strictures, these periductal cancers may be very small and thus, beyond the resolution of standard imaging techniques. Whether a bile duct stricture harbors an underlying malignancy or not in the presence of stricturing conditions such as PSC remains a pertinent issue for current technologies (9).

CCA has hitherto been a tumor with an extremely high mortality, unless operable surgically, but new targeted chemotherapy techniques offer promise (10). The need for accurate and early diagnosis has not therefore been greater.

## 3 The approaches to the problem

Resolution in MRI is determined by signal-to-noise ratio (SNR), with higher SNR allowing smaller voxels and higher resolution. However, SNR is limited by body noise using an external radiofrequency (RF) coil such as a “torso array” configuration (11). One solution may be intraductal MRI, with detection carried out using a catheter receiver inserted into the common bile duct at endoscopic retrograde cholangiopancreatography (ERCP). Internal RF coils offer locally increased SNR by virtue of their non-uniform signal reception, which reduces the field-of-view (FOV) for body noise (12). Higher SNR can then be obtained near the coil, but this reduces as the reciprocal of radial distance squared for rectangular RF coils, so a catheter receiver can outperform an external coil over a cylindrical volume coaxial to a duct. The local SNR advantage of internal coils in endoluminal MRI has been verified by many authors, for example in arterial imaging (13), gastrointestinal imaging (14), and endoscopy (15), and internal RF coils have already been used in biliary drainage tubes (16). Non-uniform reception complicates image interpretation but can be compensated by using relaxometry (17), estimating parameters by nonlinear least-squares fitting (18) and plotting spatial variations of time constants rather than grey scale images.

Parametric mapping of liver disease has already been demonstrated with external coils (19, 20), and we have previously reported methodology for intraductal MRI. Work has focused on duodenoscope modification for MR environments (21), the production of catheter-based receivers (22), the verification of the local SNR advantage of catheter receivers over torso array coils (23) and initial *ex vivo* imaging studies of surgical specimens (24).

Receivers have been constructed from thin-film circuits formed in copper-clad Kapton and mounted on tubular scaffolds using heat-shrink tubing. Division of the circuit into arrays of magnetically coupled, figure-of-eight-shaped L-C resonators can reduce coupling to B1 magnetic and corresponding electric fields during excitation (25), but this leads to a segmented FOV. The catheters are flexible and compatible with biopsy channels and guidewires. Technical challenges included developing the design concept, simulating electro-magnetic performance, integrating flexible circuits on catheters, connecting receivers to auxiliary coil interfaces, and performing initial assessments of RF heating potential. Imaging has been carried out using a clinically available whole-body 3T Philips Achieva™ (Philips, Best, the Netherlands) MRI system in Khon Kaen in North-East Thailand, the epicenter of liver fluke-associated cholangiocarcinoma (24). Clinical challenges included synchronizing imaging with surgery in a busy hospital, identifying specimens suitable for cannulation, and correlating T2 maps of CCA with histopathology for the first time. We explain below the principle of and make the case for intraductal T2 mapping.

## 4 The potential solution

The *ex vivo* intraductal imaging shown in the diagrams was performed on resection specimens from Thai patients with CCA at Khon Kaen University Hospital (KKUH), following the granting of ethics approval by the local Ethics Committee (Ref. HE581409) and the provision of prior written informed consent from patients. The imaging was conducted according to the ethical precepts set out in the Declaration of Helsinki of 1975.

**Figure 1:**
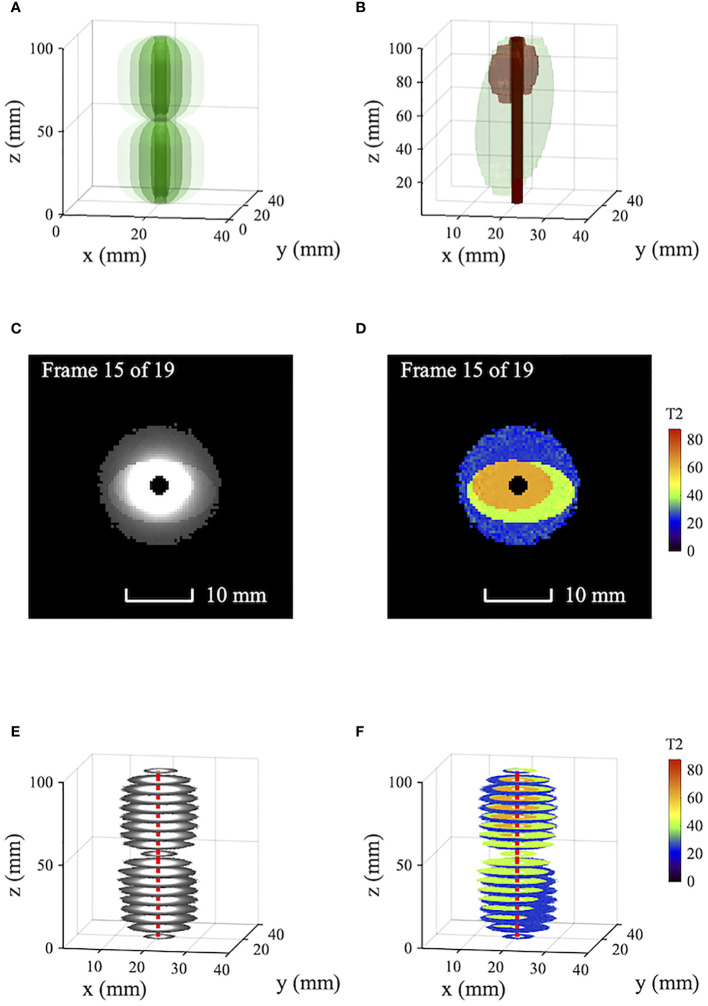
Simulated T2-weighted axial images and T2 maps of model tissue obtained using a catheter receiver: **(A)** Receiver tip reception pattern; **(B)** model tissue showing periductal fibrosis and tumor; **(C)** T2-weighted images; **(D)** T2 maps; **(E, F)** volumetric data. Blue - liver parenchyma; green - fibrosis; Brown - tumor; red - catheter track.

**Figure 1** compares simulated T2-weighted images and T2 maps of model tissue. **Figure 1A** shows the signal reception pattern of a 3*mm* diameter catheter receiver with 1/*r* ^2^ radial sensitivity variation and 50 *mm*-long segments, highlighting the first two sensitive lobes; other lobes are similar. **Figure 1B** shows the model tissue, assumed roughly coaxial to a central receiver. Two artificially homogeneous ellipsoidal tissue volumes are shown, with *T*2 values of 63 ms (brown, representing tumor tissue) and 42 ms (green, representing periductal fibrosis). Surrounding tissue (liver parenchyma, not shown) has a *T*2 of 28 ms. Imaging and T2 mapping are simulated using a single echo time *TE*_1_=140/9*ms* and five echo times *TE_n_* =*n*×140/5*ms*, respectively. T2 values are estimated using a least-squares non-linear fit to mono-exponential decay without bias correction (18). A peak signal-to-noise ratio (SNR) of 800 is assumed at the catheter. Cropping at a low signal level is used to avoid presenting noise beyond the limit of effective reception.

**Figures 1C, D** show axial T2-weighted images and T2 maps obtained by simulation. **Figures 1E, F** show corresponding volumetric data. In the images, the signal variation introduced by non-uniform reception is so large that little can be seen of the important tissue boundaries; the reception pattern of the catheter is dominant. While the sensitivity variation can be compensated, the catheter axis must be accurately known, and the procedure is slow. Tissue differentiation is obtained from the T2 maps directly, which clearly show tumor (brown), fibrosis (green) and parenchyma (blue). The effect of radially decreasing SNR is to increase the scatter and bias of estimated *T2* values (18). However, near the catheter, homogeneity is high and tissue boundaries are sharp.

**Figure 2:**
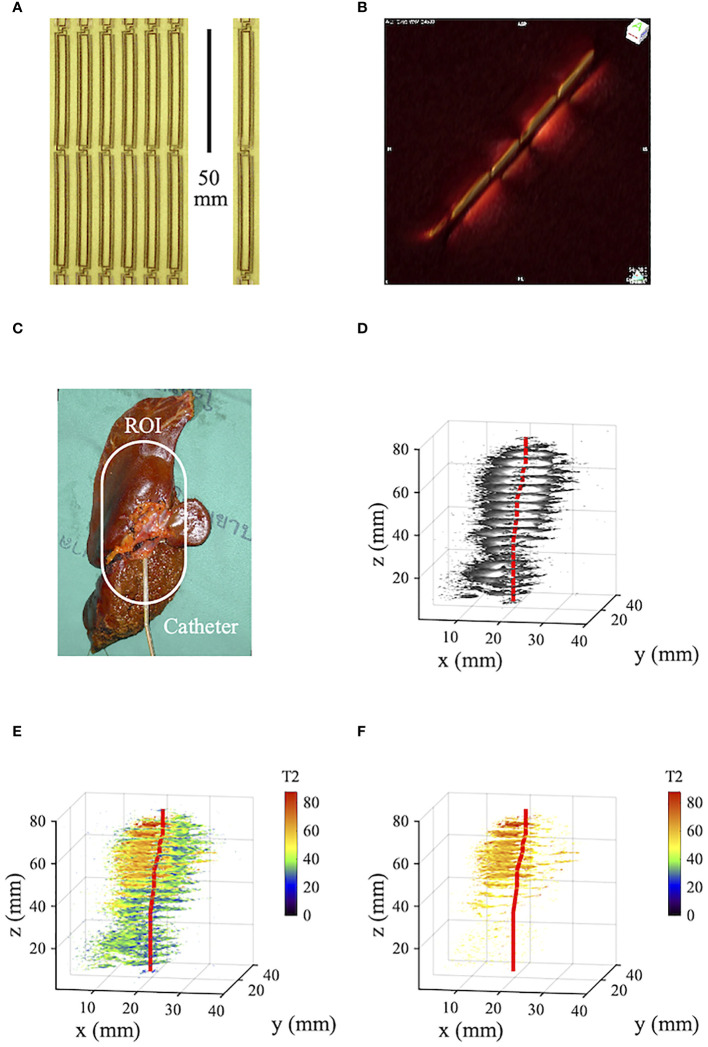
**(A)** Panel and single element of thin-film receiver circuits; **(B)** catheter reception pattern; **(C)** human tissue specimen with intraductal CCA; **(D)** volumetric T2-weighted imaging data; **(E)**, volumetric relaxometry data; **(F)** as **(E)** but excluding T2 values below 45. Blue - liver parenchyma; green - periductal fibrosis; yellow - original duct; brown - tumor; red - catheter track.

**Figure 2** shows data from an *ex vivo* resection specimen from a Thai patient with polypoid intraductal CCA, adapted and modified from a previous publication (24). Imaging was carried out at 3T using a catheter receiver on a resection specimen immediately post-surgery. **Figure 2A** shows part of a much longer panel of thin-film receiver circuits and a single circuit before mounting on a catheter. The circuits are devoid of protrusions, and each figure-of-eight loop has a half-length of 50 *mm*. **Figure 2B** shows the segmented signal reception pattern, obtained by imaging tank phantoms on either side of the catheter and plotting coronal scans as surface-rendered volumetric data. Multiple sensitive lobes can be seen, each again 50 *mm* long. **Figure 2C** shows the specimen with the catheter inserted into a visibly enlarged duct in segment 2. The region of interest (ROI) is highlighted. **Figure 2D** shows cropped axial T2-weighted volumetric images obtained using a spin-echo sequence with *TE*=9 *ms*. As with **Figure 1E**, this presentation mainly highlights the catheter location and the first one and a half lobes of the segmented receiver reception pattern. **Figure 2E** shows corresponding T2 maps, obtained from five excitations with *TE* between 9*ms* and 95 *ms*. The tissue is well differentiated, and the assigned *T*2 values were verified by histopathology (24). Resolution is high, and CCA-induced thickening of the duct wall is apparent; with the exclusion of T2 values below 45*ms* (**Figure 2F**), the tumor boundary is clear and correlated well with **Figure 2C**.

The T2 maps presented here have been compared with axial MR images obtained using external and internal coils (24). In the former case, the FOV encompasses the entire torso, and contrast-enhanced imaging allows rapid location of strictures and trapped bile *in vivo*. However, early duct wall changes and the full extent of periductal and intraductal tumors are insufficiently conspicuous. In the latter case, the FOV is limited to the immediate vicinity of the catheter, and uncorrected images cannot easily be interpreted. Duct wall enlargement is clarified after correction for non-uniform signal reception, but the procedure for optimization of the correction center is lengthy and may not be practical for oblique or curved catheter tracks.

Intraductal T2 mapping does appear to offer advantages. However, implementation may require clinic layout alterations, including co-location of endoscopy and MRI suites and provision of facilities for cleaning or disposal of catheter receivers. In addition, anesthesia and equipment for respiratory gating and software for motion tracking would be needed for patients deemed incapable of breath-holding.

## 5 Discussion

Few alternative imaging techniques are emerging for CCA, and all suffer from limitations (26). Established X-ray fluoroscopy visualizes stricture-induced dilation rather than duct wall changes. The FOV of endoscopic camera probes is restricted to the biliary mucosal layer. Contrast-enhanced CT suffers from relatively low resolution and soft-tissue contrast. Trans-abdominal and intraductal ultrasound also suffer from low resolution and contrast, but the former provides an effective mass screening tool (27). While molecular and nuclear imaging techniques hold theoretical promise, to date, positron emission tomography (PET) and single-photon emission computed tomography (SPECT) offer low resolution, unless combined with MRI or CT (9).

Questionable aspects for future adoption of intraductal MRI include the safety, complexity and cost of a procedure involving both ERCP and MRI and the resolution enhancement obtained *in vivo*. ERCP is routinely used to clarify difficult CCA cases, but MR-compatible endoscopic procedures under sedation would be required for patient safety. Cannulation of the ducts of interest might be difficult if strictures are present. Though additional scans are required for T2 mapping, and off-axis coil orientations will reduce the local SNR advantage, recent technical advances in imaging methodology, including artificial intelligence, can help to leverage the information available and mitigate the challenges in motion and SNR (28, 29). The radial FOV of the catheter receiver is limited, but may be sufficient for early-stage disease, and extension of the axial FOV along the entire catheter length (**Figure 2B**) allows catheter tracking. Necessary hardware has been demonstrated, but manufacture of disposable receivers would be needed, together with non-magnetic duodenoscopes, unless these are withdrawn before imaging, or the catheter is introduced via percutaneous transhepatic cholangiogram (PTC). Interpretation of T2 maps would require a comparison database but may reveal early-stage tissue changes not visible on contrast-enhanced MRI. Potential benefits include more effective surgical planning and improved determination of R0 margins. Both might increase the survivability of CCA, currently at a very low level.

Potential barriers to adoption include additional equipment, consumable and procedure costs, and the need for training in catheter receivers and T2 mapping. Additional expenses will be mitigated by the reduction in care costs expected from increased surgical success rates. Training for endoscopists will be similar in scope to the requirements for camera probes, while training for radiographers and radiologists may amount to revision of earlier specialized courses. Infrastructure changes have been mentioned above and would be considered on a clinic-by-clinic basis.

Further pilot studies are warranted to assess *in vivo* feasibility, but the potential for increased and earlier tumor detection seems promising, particularly in populations such as in Thailand where CCA is not uncommon and underlying causes are understood, resulting in a need for screening programs (1). Furthermore, T2 mapping with standard external phase-array RF coils, rather than the intraductal RF coils we describe here, may provide a simpler method of identifying enlarged bile ducts, albeit at lower image resolution. This may be possible because of the strong differentiation between liver parenchyma and ductal tissue. The same principles may also be useful for better and earlier assessment of hepatocellular carcinoma, particularly in the context of a nodular, cirrhotic liver.

## Data availability statement

The raw data supporting the conclusions of this article will be made available by the authors, without undue reservation.

## Ethics statement

The studies involving humans were approved by Khon Kaen University Ethics Committee. The studies were conducted in accordance with the local legislation and institutional requirements. The participants provided their written informed consent to participate in this study.

## Author contributions

RS: Conceptualization, Data curation, Formal analysis, Investigation, Methodology, Project administration, Software, Supervision, Writing – original draft, Writing – review & editing. CW: Data curation, Formal analysis, Investigation, Validation, Writing – review & editing. EK: Formal analysis, Investigation, Methodology, Software, Validation, Writing – review & editing. AT: Investigation, Methodology, Project administration, Resources, Visualization, Writing – review & editing. WB: Data curation, Investigation, Validation, Writing – review & editing. PS-N: Formal analysis, Investigation, Methodology, Project administration, Resources, Supervision, Writing – review & editing. SZ: Methodology, Validation, Writing – review & editing. ST-R: Conceptualization, Funding acquisition, Project administration, Supervision, Writing – original draft, Writing – review & editing. NC: Investigation, Methodology, Resources, Supervision, Writing – review & editing. WL: Data curation, Formal analysis, Investigation, Project administration, Resources, Writing – review & editing.
